# The diagnostic significance of blood-derived circRNAs in NSCLC: Systematic review and meta-analysis

**DOI:** 10.3389/fonc.2022.987704

**Published:** 2022-10-24

**Authors:** Weijie Yu, Ruixue Liu, Zhoulin Miao, Liwei Zhang, Ilyar Sheyhidin, Julaiti Ainiwaer

**Affiliations:** ^1^ Department of Cardio-Thoracic Surgery, The Fourth Affiliated Hospital of Xinjiang Medical University, Xinjiang, China; ^2^ Department of Thoracic Surgery, The First Affiliated Hospital of Xinjiang Medical University, Xinjiang, China; ^3^ Radiology Department, Qingdao Municipal Hospital, Qingdao, Shandong, China

**Keywords:** circRNAs, NSCLC, diagnosis, blood-derived, oncology

## Abstract

**Background:**

Using blood-derived circular RNAs (circRNAs) may be an efficient tool for noninvasive fluid biopsy in diagnosing non-small cell lung cancer (NSCLC). However, no relevant systemic meta-analysis has been conducted so far to support the diagnostic value of using blood-derived circRNAs in NSCLC clinically. The aim of this study is to clarify the issue through a meta-analysis.

**Methods:**

A systematic search strategy was used to search relevant literature in the databases of PubMed, Web of Science, and Cochrane Library from 2017 to 2022. The relationship between the diagnostic accuracy of circRNAs and NSCLC was analyzed. For the purpose of evaluating the quality of the literature, Quality Assessment of Diagnostic Accuracy Studies-2 (QUADAS-2) was used. Statistical analyses were assessed using Stata software (version 17.0) and META-DISC (version 1.4).

**Results:**

The meta-analysis included 1,093 patients with NSCLC and 959 controls. Results are as follows: pooled sensitivity, 0.78 (95% CI = 0.71–0.83, I^2^ = 71.86); pooled specificity, 0.76 (95% CI = 0.70–0.82, I^2^ = 70.12); pooled positive likelihood ratio (PLR), 3.3 (95% CI = 2.6–4.2, I^2^ = 37.56); pooled negative likelihood ratio (NLR), 0.29 (95% CI = 0.23–0.37, I^2^ = 64.67); diagnostic odds ratio (DOR), 11.42 (95% CI = 7.88–16.56, I^2^ = 99.05); area under the receiver operating characteristic curve (AUC), 0.84 (95% CI = 0.80–0.87). Based on the subgroup analysis, it appears that the heterogeneity is primarily caused by the NSCLC subgroup.

**Conclusion:**

circRNAs are highly useful diagnostic biomarkers for NSCLC in China. Further prospective studies on the diagnostic value of circRNAs should be conducted in multiple countries.

**Systematic review registration:**

https://www.crd.york.ac.uk/prospero/, identifier CRD42022323804.

## Background

Circular RNAs (circRNAs) are a class of circular non-coding RNAs that are covalently formed by reverse 3′ and 5′ clipping (1). The original circRNAs were first located in the cytoplasm of HeLa cells in eukaryotic cells and then gradually found in humans and animals (2). This discovery has evolved into a current research hot spot. However, the initial understanding was not sufficient, and researchers once considered it as an error product in the transcription process (3). Until the emergence and development of high-throughput sequencing technology, researchers shifted their insights into circRNAs and gradually deepened their functional exploration. Now, circRNAs have been confirmed to play an essential role in the sponge adsorption of microRNAs (miRNAs), gene regulation of transcription, and protein translation (4–8). In addition, circRNAs are closely related to many malignant tumors, such as lung cancer and gastric cancer (9, 10). The abnormal expression of circRNAs also plays a vital role in the regulation of tumor cell invasion, metastasis, apoptosis, immune escape, etc. and is a promising biomarker and therapeutic target today (11–13). Moreover, circRNAs also have many characteristics. Due to their unique structure, circRNAs are not susceptible to ribonucleases, which provides them with strong structural stability. circRNAs have been demonstrated to be obtained from various sources, including tissues, and enter into the bloodstream by the exosomes. Aside from blood, urine and saliva can also be obtained. However, their expression varies by disease and tissue, indicating that they are tissue-specific (14, 15). It is also due to the characteristics of circRNAs that ensure circRNAs can be detected in blood as tumor markers, which provides a new direction for liquid biopsy in disease diagnosis (16, 17).

Lung malignancies represent a considerable proportion of today’s tumor-related disease spectrum. In 2018, there were 2.1 million new cases of lung cancer worldwide, accounting for 11.6% of new tumor cases, and 1.8 million people died of lung cancer, accounting for 18.4% of cancer-related deaths (18). Moreover, non-small cell lung cancer (NSCLC) accounts for approximately 80%. A vast number of such cases are not only due to environmental factors but also due to the lack of obvious and specific symptoms of the disease, which make it difficult to attract the attention of patients, so they miss the best opportunity for treatment. Achieving early detection and early diagnosis to get medical treatment is significant for patients with NSCLC. It is widely regarded that the efficacy of tumor markers in NSCLC remains controversial, although blood-derived tumor markers such as carcinoembryonic antigen (CEA), neuron-specific enolase (NSE), cytokeratin 19 fragment antigen21 -1 (CYFRA21-1) have been listed as recommended tumor markers by many countries.

Huang et al. (19) explored the diagnostic efficacy of blood-derived hsa_circ_0070354 for NSCLC and found that it was superior to traditional tumor markers in the diagnosis. Wang et al. (20) evaluated the diagnostic performance of circRNAs for lung cancer by summarizing tissue-derived circRNAs, but in clinical practice, tissue must be obtained by puncture or surgical resection, which is meaningless for body fluid diagnosis. Therefore, high-level evidence of blood-derived circRNAs in the diagnosis of lung cancer is currently required.

In summary, this paper will explore the diagnostic value of blood-derived circRNAs in NSCLC and summarize the mechanism of the literature to provide new directions for clinical work and future scientific research.

## Materials and methods

This meta-analysis was performed following the Preferred Reporting Items for Systematic reviews and Meta Analyses (PRISMA) statement.

### Registration

The study has been registered on the PROSPERO website (ID: CRD42022323804).

### Data source and search strategy

The electronic databases, including PubMed, Web of Science, Cochrane Library, are used to systematically search articles from January 2017 to January 2022. The following search terms or keywords are used: (“circRNAs or circular RNAs”) and (“NSCLC or non-small cell lung cancer or Carcinoma, Non-Small Cell Lung or Lung squamous cell carcinoma, Adenocarcinoma, Lung or lung adenocarcinoma or Carcinoma, Non Small Cell Lung or Carcinomas, Non-Small-Cell Lung or Lung Carcinoma or Non-Small-Cell, Lung Carcinomas or Non-Small-Cell Lung Carcinomas or LCLC or large cell lung cancer”) and (“biomarkers”) and (“diagnosis”) and (“serum or blood or plasma”). Some articles were manually searched for completeness and comprehensiveness. Two researchers (WY and RL) read titles, abstracts, and full-text articles to choose the right article. Reference lists of articles were reviewed and retrieved to further identify potentially relevant studies.

### Inclusion and exclusion criteria

Inclusion criteria include the following: 1) studies analyzing the relationship between circRNAs and diagnosis of NSCLC; 2) studies providing data on the sensitivity (SEN), specificity (SPE), true positive (TP), false positive (FP), true negative (TN), or false negative (FN); 3) All standard evidence must be the pathology; 4) Source of the circRNAs is blood-based including serum and plasma; 5) None of the included patients had a prior history of other cancers or metastatic cancer from other sites or had received chemotherapy or radiotherapy prior to plasma collection.

The exclusion criteria include the following: 1) repetitive research; 2) letters, editorials, commentaries, or abstracts; 3) studies involving ineligible patients or controls; 4) studies lacking data; 5) studies in a non-English language; 6) Source of the circRNAs is from tissue or other types; 7) In the absence of a control group research; 8) There was no comparative study. If the results or research cases are overlapped, only the first study or the complete study was included; 9) Patients had a prior history of other cancers or metastatic cancer from other sites or had received chemotherapy or radiotherapy prior to plasma collection.

### Data extraction and quality assessment

Two reviewers (WY and RL) extracted the data. If necessary, the discrepancies were solved by the third reviewer (ZM). The following information is extracted from each article: first author name, publication time, area, impact factor, number of case, circRNA type, sample type or origination, cancer type, sensitivity, specificity, area under the receiver operating characteristic curve (AUC), testing method and differential expression of circRNAs. If the value of TP, FP, TN, or FN is omitted in the article, four indexes will be calculated from the sensitivity, specificity, and number of cases.

### Study quality assessment

The Quality Assessment of Diagnostic Accuracy Studies-2 (QUADAS-2) Evaluation Scale was used by two reviewers independently to assess the quality of every included article. A score beyond 9 is deemed as good quality.

### Statistical analysis

The STATA 17.0 is used to analyze the diagnostic value of circRNAs. The sensitivity [TP/(FN+TP)], specificity [TN/(FP+TN)], positive likelihood ratio (PLR) [sen/(1-spe)], negative likelihood ratio (NLR) [(1-sen)/spe], diagnostic odds ratio (DOR) [PLR/NLR], 95% confidential intervals (95% CIs), summary receiver operating characteristic (SROC)curve, and AUC were plotted to estimate the diagnostic value of circRNAs. A two-sided p < 0.05 was considered statistically significant. A fixed-effects model is used if the heterogeneity is minimal (I^2^ < 50%); otherwise, a random-effects model was used in the significant heterogeneity (I^2^ > 50%). The possible source of heterogeneity is performed by threshold effect, regression analysis, subgroup analysis, and sensitivity analysis. The value of RDOR is calculated by the META-DISC 1.40.

## Results

### Search selection and characteristics

**Figure 1:**
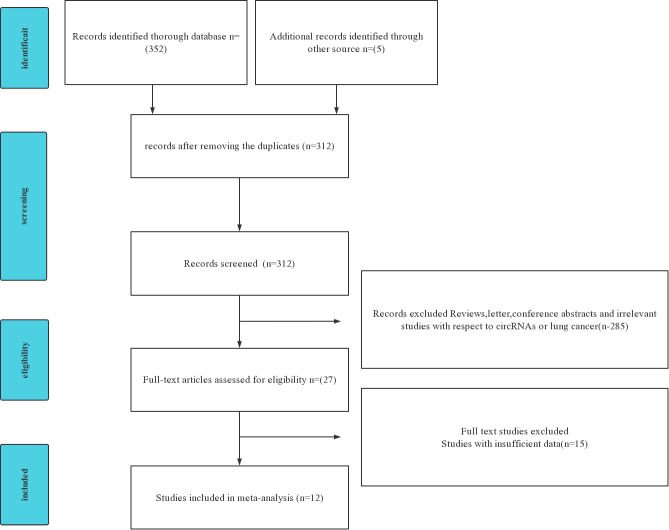
Preferred Reporting Items for Systematic reviews and Meta Analyses (PRISMA) flowchart of the included and excluded study selection process.

The flow diagram for literature research processes is shown in **Figure 1**. circRNAs derived from the blood are analyzed to evaluate the diagnostic value of NSCLC. The characteristics of the included studies are summarized (**Table 1**). A total of 1,093 patients with NSCLC and 959 controls, who are all from Asia, were analyzed in this article with 12 articles and 12 kinds of circRNAs. All circRNAs are detected in qRT-PCR.

**Table 1:** Characteristics of circRNAs and quality of included studies in diagnosing.

First author	Country	Year	IF	circRNAs	Cancer type	circRNA regulation	Sample type	Sample size		Testing method	I–II/III–IV	TP	FP	FN	TN	Quality score
								No. of Cases	No. of control							
Guo-jun Lu (21)	Asia(China)	2020	4.147	hsa_circ_0001715	LA	Up	Plasma	60	57	qRT-PCR	31/29	53	16	7	41	9
Xiaoli Zhu (22)	Asia(China)	2017	5.542	hsa_circ_0013958	LA	Up	Plasma	30	30	qRT-PCR	-/-	20	2	10	28	9
Fan Yang (23)	Asia(China)	2021	3.269	circRNA_001846	NSCLC	Up	Serum	206	206	qRT-PCR	109/97	161	39	45	167	10
Chengzhi Ding (24)	Asia(China)	2020	4.599	circ-MEMO1	NSCLC	Up	Serum	30	25	qRT-PCR	-/-	17	1	13	24	9
Jie Shi (25)	Asia(China)	2021	11.61	circ-pvt1	LA	Up	Serum	104	110	qRT-PCR	50/54	95	43	9	67	9
Xiuyuan Li (26)	Asia(China)	2018	5.5	CircPVT1	NSCLC	Up	Serum	45	45	qRT-PCR	-/-	32	9	13	36	10
Xiaoxia Liu (27)	Asia(China)	2019	5.531	hsa_circ_0086414	LA	Down	Plasma	54	54	qRT-PCR	-/-	42	18	12	36	9
				hsa_circ_0005962	LA	Up	Plasma	54	54	qRT-PCR	-/-	39	15	16	39	9
Yong Zhou (28)	Asia(China)	2022	2.718	hsa_circ_0072309	NSCLC	Up	Serum	46	40	qRT-PCR	26/20	40	7	6	33	10
Dong Hang (29)	Asia(China)	2018	4.452	circFARSA	NSCLC	Up	Plasma	50	50	qRT-PCR	–	32	17	18	33	9
Ziyi Peng (30)	Asia(China)	2021	3.786	CircTOLLIP	NSCLC	Down	Blood	88	76	qRT-PCR	–	66	26	22	50	10
Yinzai He (31)	Asia(China)	2021	3.25	circ_0048856	NSCLC	Up	Serum	50	50	qRT-PCR	–	44	10	6	40	10

circRNA, circular RNA; NSCLC, non-small cell lung cancer; AUC, area under the receiver operating characteristic curve; LA, lung adenocarcinoma; FN, false negative; FP, false positive; TN, true negative; TP, true positive; qRT-PCR, quantitative real-time polymerase chain reaction.

All of the studies are independently scored based on QUADAS-2 score system.

### Results of the meta-analysis

**Figure 2:**
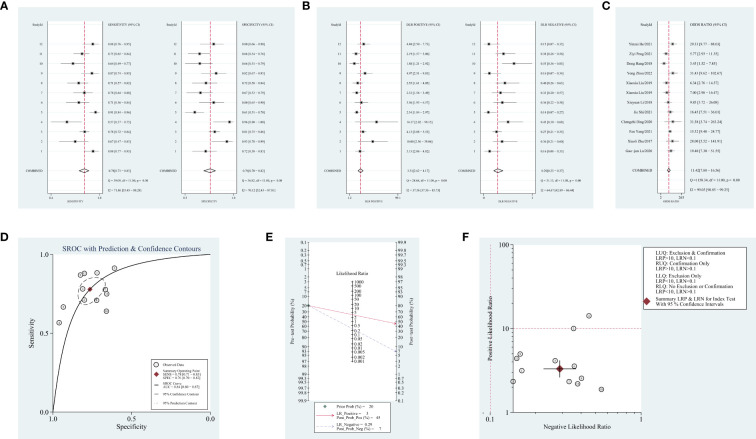
Forest plots of diagnostic accuracy index and summary receiver operating characteristic (SROC) curve and Fagan’s nomogram for likelihood ratios, a likelihood ratio scattergram, publication bias. **(A)** Forest plots of sensitivity and specificity for circular RNAs (circRNAs) in diagnosis. **(B)** Forest plots of the positive likelihood ratio and negative likelihood ratio in diagnosis. **(C)** Forest plots of the diagnostic odds ratio in diagnosis. **(D)** SROC curve for circRNAs in diagnosing non-small cell lung cancer (NSCLC). **(E)** Fagan’s nomogram for likelihood ratios. **(F)** A likelihood ratio scattergram.

Obvious heterogeneity was assessed using the random-effects model (I^2^ > 50%). For the value of blood-derived circRNAs in diagnosing NSCLC, the result are as follows: pooled sensitivity, 0.78 (95% CI = 0.71–0.83, I^2^ = 71.86); pooled specificity, 0.76 (95% CI = 0.70–0.82, I^2^ = 70.12); pooled PLR, 3.3 (95% CI = 2.6–4.2, I^2^ = 37.56); pooled NLR, 0.29 (95% CI = 0.23–0.37, I^2^ = 64.67); DOR, 11.42 (95% CI = 7.88–16.56, I^2^ = 99.05); AUC, 0.84 (95% CI = 0.80–0.87). Forest plots and SROC curves are shown in **Figures 2A–D**. As shown in **Figure 2E**, the result shows a pretest probability of 20%, and the posttest probability rates, given the positive and negative results, were respectively 45% and 7%. Among the 12 included studies (one article contains two types of circRNAs), 11 studies are located in the right lower quadrants, implying that the circRNAs are valuable in the diagnosis (**Figure 2F**).

### Threshold effect

The threshold effect was calculated using the Spearman rank correlation. The Spearman correlation coefficient was 0.354 (p = 0.259), which suggests that the heterogeneity is not caused by the threshold effect.

### Meta regression analysis

Because of the value of I^2^ >50%, it suggests evident heterogeneity. From the baseline of study characteristics, cancer type, sample type, and expression situation may be the potential reasons for the heterogeneity. So, we further completed the meta-regression analysis. No significant causes of heterogeneity were found (p > 0.05). The results are shown in **Table 2**.

**Table 2:** Meta regression analysis.

	Coeff	Std.Err	RDOR	P value	95% CI
Cancer type	-0.2529914	0.1375258	0.71	0.103	(-0.570126 to 0.0641436)
Sample type	0.3997855	0.2138254	1.95	0.098	(-0.093296 to 0.8928676)
Expression situation	-0.5589196	0.2791767	0.34	0.080	(0.820598–2.624503)

### Sensitivity analysis

**Figure 3:**
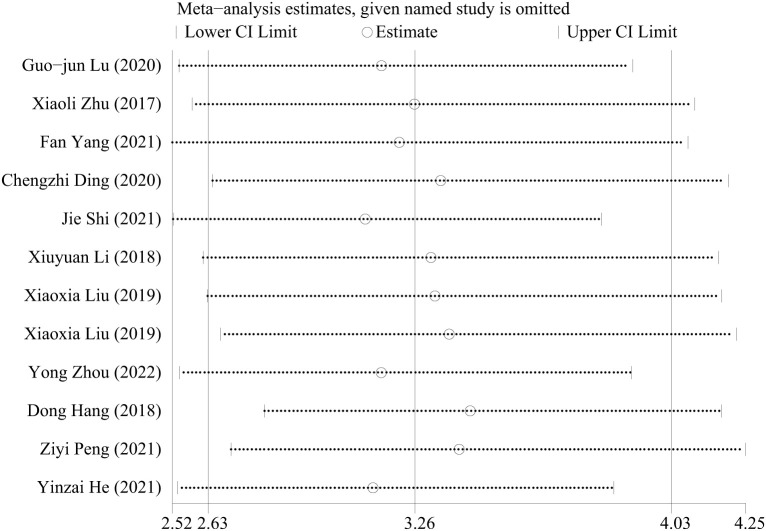
Sensitivity analyses for diagnosis analysis.

The value of I^2^ >50% suggests apparent heterogeneity. Sensitivity analysis is further completed to discover the heterogeneity. The following chart conveys a conclusion that although there are some inconsistencies when successfully omitting one in the literature, the result is robust (**Figure 3**).

### Subgroup analysis

In order to find out the reason for heterogeneity, a subgroup analysis was performed based on the cancer type, sample type, and expression condition. The AUC values for all three subgroups are >0.8, suggesting an excellent diagnosis value. Aside from the NSCLC subgroup, almost all of the subgroup analyses suggest that the I^2^ of the subgroup is low (I^2^ <50%). So, these subgroups may cause the heterogeneity. Moreover, results show that the diagnosis effectiveness of serum-based circRNAs is greater than that of the plasma-based circRNAs. Because of the paucity of literature on whole blood-based circRNAs, it was impossible to calculate I^2^ and p values. So do downregulated circRNAs (**Table 3**).

**Table 3:** Subgroup analysis of NSCLC diagnosis.

	Number of studies	I^2^	p value	Sensitivity	Specificity	PLR	NLR	DOR	AUC
Cancer type LA NSCLC									
	5	46.8%	0.111	0.81	0.72	2.9	0.27	11	0.82
	7	64.3%	0.01	0.76	0.78	3.5	0.31	11.24	0.84
Sample type Plasma Serum Blood									
	5	48.9%	0.098	0.75	0.73	2.8	0.35	7.99	0.80
	6	42.1%	0.124	0.8	0.8	4.1	2.4	17	0.87
	1	–	–	0.9	0.902	9.183	0.111	82.837	0.95
Expression regulation									
Up	10	59.2%	0.009	0.77	0.80	3.9	0.28	14	0.86
Down	2	–	–	0.76	0.66	2.25	0.36	6.21	–

LA, lung adenocarcinoma; NSCLC, non-small cell lung cancer; PLR, positive likelihood ratio; NLR, negative likelihood ratio; DOR, diagnostic odds ratio; AUC, area under the receiver operating characteristic curve.

Publication bias was assessed in the end.

### Publication bias

**Figure 4:**
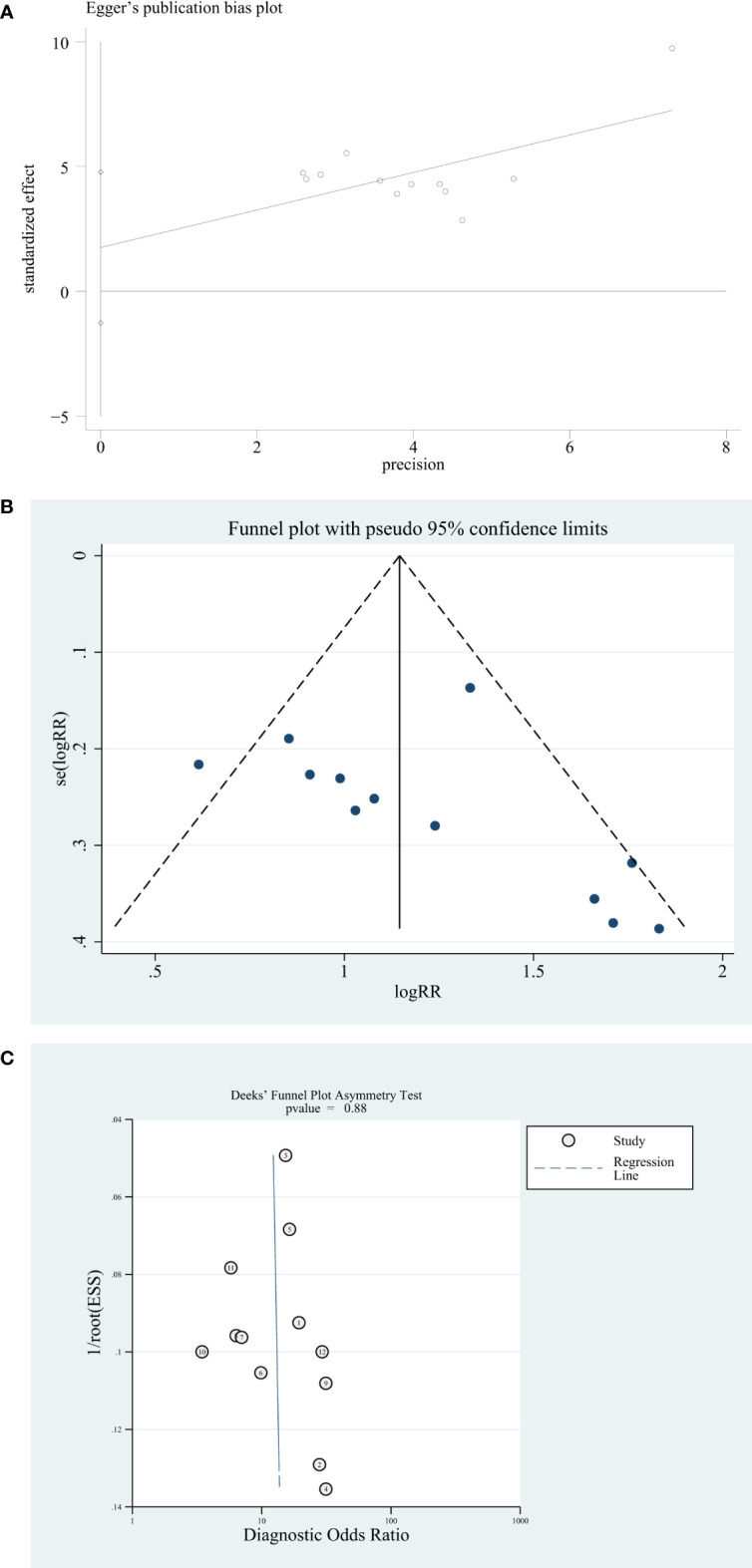
Publication bias analysis. **(A)** Egger’s publication bias plot. **(B)** Funnel plot. **(C)** Deeks’ funnel plot.

The publication bias of the included studies was certified by STATA 17.0 software. The Egger test and funnel plot are used to assess the publication bias. The result of the Egger test shows 0.027 (p > 0.05) (**Figure 4A**). The funnel plot was constructed with the STATA 17.0 software, and the funnel plot is symmetrical and shows no publication bias (**Figure 4B**). Deeks’ funnel plot is also used to evaluate the publication bias, and the result shows 0.88 (p > 0.06). So, Deeks’ funnel plot shows no publication bias (**Figure 4C**).

## Discussion

Nowadays, the discovery of various biomarkers and the exploration of body fluid diagnosis have gradually deepened. With the recognition of circRNAs, many kinds of circRNAs are explored, and mechanisms of diagnosis are identified (32, 33). Researchers know the importance of circRNAs. According to existing research, tissue-derived RNA has better diagnostic performance, but obtaining tissue means trauma to the patient. Nowadays, liquid biopsy in a non-invasion form is deemed a revolutionary tool in the diagnosis and has attracted much attention worldwide. As an excellent biomarker for diagnosis, it must hold several characteristics such as analytical validity (34), clinical validity, and utility. Combining the advantages of circRNAs, it may be a promising biomarker to distinguish normal, benign disease from malignant tumor. However, a blood-based meta-analysis of circRNAs with respect to diagnosis is absent. This article makes a supplement to this field and expresses the significance of circRNAs as biomarkers in liquid biopsy. A total of 12 articles are enrolled from 2017 to 2022, containing 12 types of circRNAs and more than 2,000 cases, in which 10 types are upregulated, and two types are downregulated. The results showed that the AUC was 0.84 (95% CI = 0.80–0.87), and the pooled sensitivity, specificity, DOR, pooled PLR, and pooled NLR were, respectively, 0.78 (95% CI = 0.71–0.83, I^2^ = 71.86), 0.76 (95% CI = 0.70–0.82, I^2^ = 70.12), 11.42 (95% CI = 7.88–16.56, I^2^ = 99.05), 3.3 (95% CI = 2.6–4.2, I^2^ = 37.56), and 0.29 (95% CI = 0.23–0.37, I^2^ = 64.67). It is generally believed that the value of AUC between 0.7 and 0.9 is considered a good diagnostic value. So, the literature indicated the significant body fluid diagnostic value of circRNAs for NSCLC. Apart from this, stability and expression of different diseases will contribute to circRNAs being a more suitable biomarker in diagnosing and solving the problem of low organ specificity of existing markers. According to existing research, the AUC of tumor tissue-based circRNAs is between 0.75 and 0.85 (34–36), which is very close to the AUC of blood-based circRNAs and means that the non-invasion blood acquisition mode in diagnosing will be adapted to apply clinically.

In addition, the result of the threshold effect calculated by the Spearman correlation coefficient shows that it is not responsible for the heterogeneity, and the outcome was 0.354 (p > 0.05). Next, the regression analysis, which involves sample type, lung cancer type, and differential expression of circRNAs, showed that these are not the reason for heterogeneity (p > 0.05). In order to find out the source of heterogeneity, results of the sensitivity analysis and subgroup analysis show that when the total cases are divided into three subgroups, the I^2^ of the LA group is less than 50% (p > 0.05). However, the I^2^ of NSCLC is greater than 50%. It means that the NSCLC subgroup might be the source of the heterogeneity. Further reading of the literature revealed that the types of disease and the case of patients in the literature might be responsible for the heterogeneity. The subgroup of serum-based circRNAs, which has an excellent AUC value, has a more extraordinary diagnosis performance than plasma-based circRNAs. Whether the difference is statistically significant is uncertain because the two subgroups contain different circRNAs and different diseases. However, Li et al. (37) believe that serum-derived RNA has higher diagnostic performance. The reason may be that serum exosomes contain a large number of stably expressed circRNAs. After dividing into three subgroups, the number of cases of some groups is fewer because some indexes cannot be calculated by the software. Using Egger test, funnel plot, and Deeks’ funnel plot to analyze publication bias, all three showed an insignificant publication bias (p > 0.05).

Similar findings have been found in a variety of malignancies, except for NSCLC. Hu et al. (38) validated that circGSK3 from plasma has a greater AUC value than 0.8 for the diagnosis of early esophageal squamous cell carcinoma (ESCC) in a cohort of 86 individuals and concluded that circGSK3β is a very excellent diagnostic marker for body fluids. Subsequent follow-up of patients in the experimental group and comparison of pre- and postoperative plasma-derived circGSK3β expression levels revealed that circGSK3β also played an excellent predictive function for postoperative recurrence and metastasis. Omid-Shafaat et al. (39) evaluated the diagnostic efficacy of circELP3 in combination with circFAF1 for breast cancer with AUC, sensitivity, and specificity of 0.891, 96%, and 62%, respectively, and concluded that it plays an excellent role as a non-invasive body fluid diagnostic marker for breast cancer. Yu et al. (40) compared plasma-derived circRNAs with α-fetoprotein (AFP) in the diagnosis of hepatitis B virus (HBV)-associated hepatocellular carcinoma and found that the combined AUC of hsa_circ_0000976, hsa_circ_0007750, and hsa_circ_0139897 was 0.843, which was much better than the AUC of 0.747 for conventional AFP. It also showed a significant effect in AFP-negative hepatocellular carcinoma. Shi et al. (41) similarly found that low expression of plasma-derived hsa_circ_001888 in gastric cancer may be a potential diagnostic marker.

To understand the role of circRNAs in the diagnosis field, the mechanism of circRNAs must be taken into consideration. Almost all abundance of circRNAs is related to disease stage and characteristics. The hsa_circ_0013958 is upregulated and promotes cell proliferation and invasion and inhibits cell apoptosis in lung adenocarcinoma. At the same time, it is also considered as a sponge of miR-134, and thus it upregulates oncogenic cyclin D1. The hsa_circ_0000190 is upregulated in the patients’ plasma with lung cancer, and it has something to do with clinical characteristics (42). The circPVT1 is upregulated, and when it is absent, the invasion or proliferation is blocked. It controls the E2F transcription factor 2 (E2F2) signaling pathway to affect NSCLC (26).

It is important to take into account several limitations in this study. First, too few samples were included. Second, the type of circRNAs and sample size are limited. It is necessary to explore more types of circRNAs. Second, this type of research contains several different types and sources of circRNAs. The number of subtypes of NSCLC also varies significantly from article to article regarding the diagnosis of this disease. These are all potential causes. Third, the evidence level of enrolled literature is different. Fourth, the research objects are in China. It is worth noting that the expression of circRNAs may differ from other countries, and application of circRNAs as biomarkers will require further study in combination with data from those countries.

## Conclusion

To sum up the above, this systematic review of data extracted from 12 theses based on patients’ blood with NSCLC indicated the significant value of circRNAs in diagnosing NSCLC. Therefore, combining the features of circRNAs, the results mean that circRNAs, especially the serum-based circRNAs, play a role in non-invasion body fluid biopsy and may open up broad prospects for specific disease diagnostics in the future, which perhaps is not limited to NSCLC, depending on its expression and organ specificity. The noninvasive method of acquiring circRNAs facilitates their wide use clinically at a lower cost, and circRNAs will be a better choice than the tumor markers. Nonetheless, better designed and larger-scale research of multinational clinical trials are required to certify the results.

## Data availability statement

The original contributions presented in the study are included in the article/supplementary material, further inquiries can be directed to the corresponding author/s.

## Author contributions

All authors contributed to the article and approved the submitted version.

## Funding

The project is supported by State Key Laboratory of Pathogenesis, Prevention and Treatment of High Incidence Diseases in Central Asia (No.SKL-HIDCA-2020-42).

## Conflict of interest

The authors declare that the research was conducted in the absence of any commercial or financial relationships that could be construed as a potential conflict of interest.

## Publisher’s note

All claims expressed in this article are solely those of the authors and do not necessarily represent those of their affiliated organizations, or those of the publisher, the editors and the reviewers. Any product that may be evaluated in this article, or claim that may be made by its manufacturer, is not guaranteed or endorsed by the publisher.
